# Identifying and Ranking Common COVID-19 Symptoms From Tweets in Arabic: Content Analysis

**DOI:** 10.2196/21329

**Published:** 2020-11-18

**Authors:** Eisa Alanazi, Abdulaziz Alashaikh, Sarah Alqurashi, Aued Alanazi

**Affiliations:** 1 Center of Innovation and Development in Artificial Intelligence Umm Al-Qura University Makkah Saudi Arabia; 2 University of Jeddah Jeddah Saudi Arabia

**Keywords:** health, informatics, social networks, Twitter, anosmia, Arabic, COVID-19, symptom

## Abstract

**Background:**

A substantial amount of COVID-19–related data is generated by Twitter users every day. Self-reports of COVID-19 symptoms on Twitter can reveal a great deal about the disease and its prevalence in the community. In particular, self-reports can be used as a valuable resource to learn more about common symptoms and whether their order of appearance differs among different groups in the community. These data may be used to develop a COVID-19 risk assessment system that is tailored toward a specific group of people.

**Objective:**

The aim of this study was to identify the most common symptoms reported by patients with COVID-19, as well as the order of symptom appearance, by examining tweets in Arabic.

**Methods:**

We searched Twitter posts in Arabic for personal reports of COVID-19 symptoms from March 1 to May 27, 2020. We identified 463 Arabic users who had tweeted about testing positive for COVID-19 and extracted the symptoms they associated with the disease. Furthermore, we asked them directly via personal messaging to rank the appearance of the first 3 symptoms they had experienced immediately before (or after) their COVID-19 diagnosis. Finally, we tracked their Twitter timeline to identify additional symptoms that were mentioned within ±5 days from the day of the first tweet on their COVID-19 diagnosis. In total, 270 COVID-19 self-reports were collected, and symptoms were (at least partially) ranked.

**Results:**

The collected self-reports contained 893 symptoms from 201 (74%) male and 69 (26%) female Twitter users. The majority (n=270, 82%) of the tracked users were living in Saudi Arabia (n=125, 46%) and Kuwait (n=98, 36%). Furthermore, 13% (n=36) of the collected reports were from asymptomatic individuals. Of the 234 users with symptoms, 66% (n=180) provided a chronological order of appearance for at least 3 symptoms. Fever (n=139, 59%), headache (n=101, 43%), and anosmia (n=91, 39%) were the top 3 symptoms mentioned in the self-reports. Additionally, 28% (n=65) reported that their COVID-19 experience started with a fever, 15% (n=34) with a headache, and 12% (n=28) with anosmia. Of the 110 symptomatic cases from Saudi Arabia, the most common 3 symptoms were fever (n=65, 59%), anosmia (n=46, 42%), and headache (n=42, 38%).

**Conclusions:**

This study identified the most common symptoms of COVID-19 from tweets in Arabic. These symptoms can be further analyzed in clinical settings and may be incorporated into a real-time COVID-19 risk estimator.

## Introduction

The ongoing COVID-19 pandemic has greatly impacted human health and well-being and has radically enforced a rigorous change in people’s lifestyles. In response to this catastrophe, we have witnessed a great effort from diverse research communities to study all aspects of this disease.

In recent years, social networks have become an important source of information where users expose and share ideas, opinions, thoughts, and experiences on a multitude of topics. Several studies have utilized the abundance of information offered by social platforms to conduct nonclinical medical research. For example, Twitter has been a source of data for many health and medical studies, such as surveillance and monitoring of flu and cancer timelines and distribution across the United States [1], analyzing the spread of influenza in the United Arab Emirates based on geotagged tweets in Arabic [2], and the surveillance and monitoring of influenza in the United Arab Emirates based on tweets in Arabic and English [3]. In addition, Twitter data have been utilized in symptom and disease identification in Saudi Arabia [4], and most recently, to examine COVID-19 symptoms as reported on Twitter [5] and to analyze the chronological and geographical distribution of infected tweeters in the United States [6].

**Figure 1:**
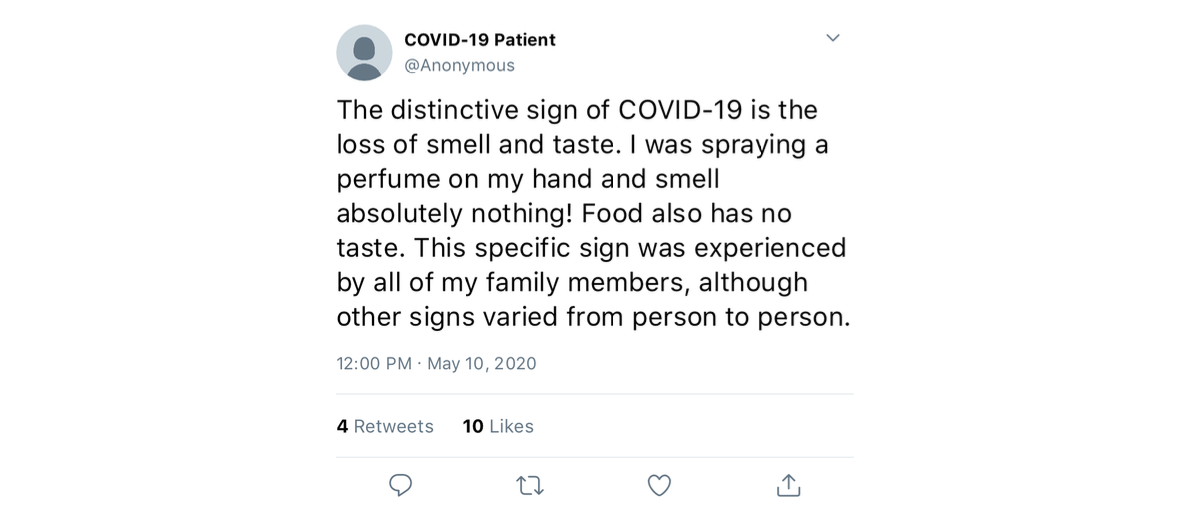
A patient with COVID-19 tweets about how the loss of smell and taste was the only common symptom across all of their family members. The tweet was anonymized and translated into English.

The Twitter platform allows researchers to obtain data on items like age, sex, geolocation, etc, along with informative posts, via data mining and analysis techniques; this can potentially result in useful insights about a specific health condition [7]. Extracting common symptoms associated with a disease from publicly available data has the potential to control the spread of the disease and identify users at high risk. It also gives new insights that call for early intervention and control. For example, Figure 1 presents the translation of a tweet (from Saudi Arabia; dating to early May 2020) that explicitly mentions the loss of smell and taste as one distinctive symptom of COVID-19. Interestingly, the official COVID-19 Questionnaire App in Saudi Arabia was updated in late May 2020 to include the sudden loss of smell and taste as one risk indicator of having COVID-19 [8]. Tracking COVID-19 symptoms in real time via public data on Twitter could have shortened the gap.

In this paper, we examined COVID-19 symptoms as reported by Arabic tweeters. First, we shuffled tweets in Arabic and searched for tweets with COVID-19 symptoms and collected tweets from users who self-reported a positive diagnosis (via clinical testing). Next, we asked infected users about the first 3 symptoms they had experienced via a voluntary survey sent through a private message.

## Methods

**Figure 2:**
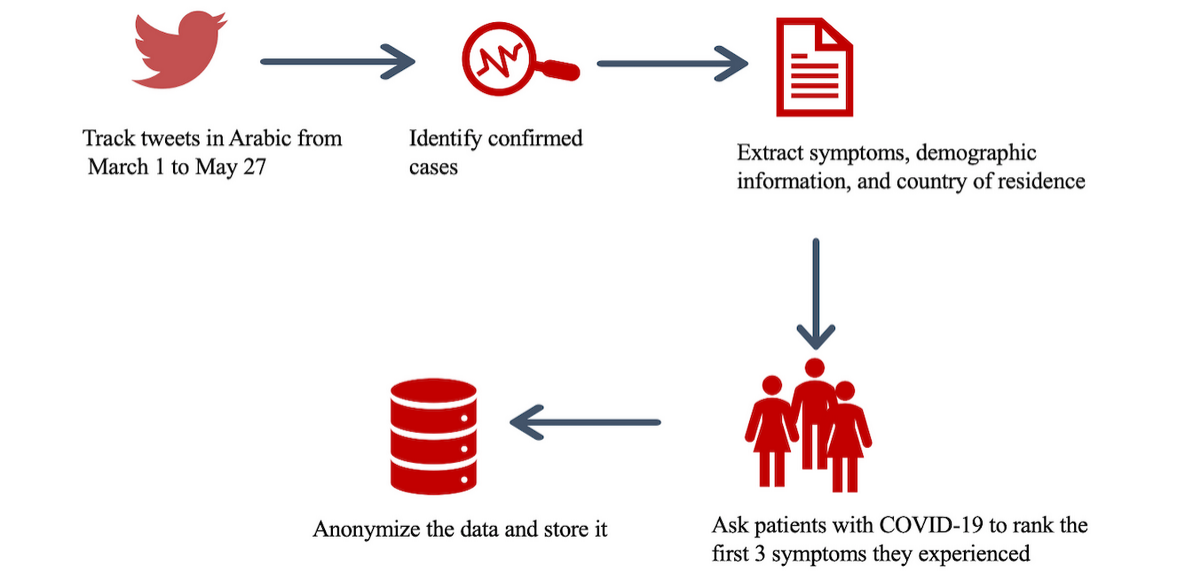
Data collection steps.

Our data collection methodology is outlined in Figure 2. First, we searched Twitter for personal reports of COVID-19 from March 1, 2020, to May 27, 2020, using 2 Arabic keywords 
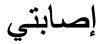
 and 
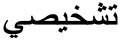
, which translate roughly to “I have been diagnosed.” Such keywords are likely to filter out reports that were not associated with a formal test result. An initial list of 463 users were collected, and 2 independent freelancers were asked to further read users’ timeline and extract symptoms that were explicitly mentioned that were related to COVID-19 and their order of appearance, if mentioned. Additional information such as user gender, date of infection, and country of residence were also collected. We assumed the date of the COVID-19 diagnosis tweet as the date of infection, if no other information was available.

In all, 270 users with COVID-19 were identified, of whom 80 shared their symptoms publicly. To further understand the chronological order of the symptoms, we asked users through Twitter personal messages to rank the first 3 symptoms they experienced right before or after testing positive for COVID-19.

We recorded the symptom ranks (from first to last) based on the received responses and publicly available data on the users’ pages. In case no order was given, an implicit order was assumed following the order in which the symptoms were mentioned by the user.

**Figure 3:**
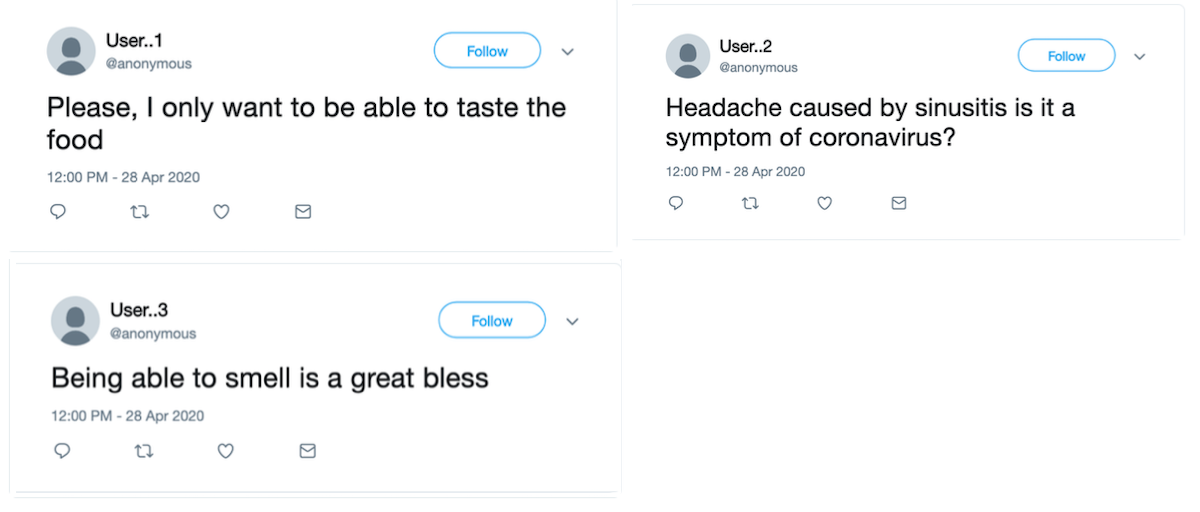
Example of tweets collected within 5 days before or after the user tweeted about having a COVID-19–positive diagnosis.

Tracking tweets containing specific keywords is not sufficient enough to obtain an overview of disease dynamics [9]. Many patients detailed their experience while infected; hence, knowing their health condition and sentiments, and tracking useful information, may lead to a better understanding of the disease symptoms. In particular, we found tweets that were posted within ±5 days of infection date to contain valuable information about early symptoms, allowing us to process and rank the symptoms. As an example, Figure 3 highlights 3 tweets by 3 different patients with COVID-19 that indirectly relay symptoms before or after being diagnosed with COVID-19. For simplicity, we set a false date (April 28, 2020) for all 3 tweets using TweetGen [10]. *User..1* tested positive on April 29, 1 day after tweeting their wish to be able to taste food; *User..2* tested positive on May 1, 3 days after complaining about a headache; and *User..3* was tested positive on April 26 and tweeted on April 28 about the loss of smell.

The examples highlighted in Figure 3 demonstrate that mining Twitter for COVID-19 symptoms requires more than a simple keyword search. In principle, the context of the tweet, as narrated by a user with COVID-19, is also important. Therefore, it is important to examine not only the tweet but also its context. To build a high-quality database of COVID-19 symptoms based on tweets in Arabic, we relied on a manual extraction of symptoms.

## Results

The majority of cases were recorded in May 2020 (n=210, 78%), followed by April (n=39, 14%) and March (n=21, 8%). This surge in May reports is understandable as most countries globally witnessed a substantial increase in the number of confirmed cases. Needless to say, some of the adopted strategies to prevent further spread of the virus (eg, active screening by the Ministry of Health in Saudi Arabia [11]) may have also helped in finding more reports in May compared to other months. We have witnessed this firsthand as some of the asymptomatic reports were mainly a result of early active screening.

Users from Saudi Arabia, Kuwait, and the United Arab Emirates constituted 85% (n=230) of reports. Nearly half of the reports came from Saudi Arabia (n=125, 46%), which is not surprising, since it is one of the top countries on Twitter with more than 15 million users [12]. Other countries (Egypt, Iraq, Bahrain, Qatar, United Kingdom, United States, Belgium, and Germany) constituted the remaining 15% (n=40).

**Figure 4:**
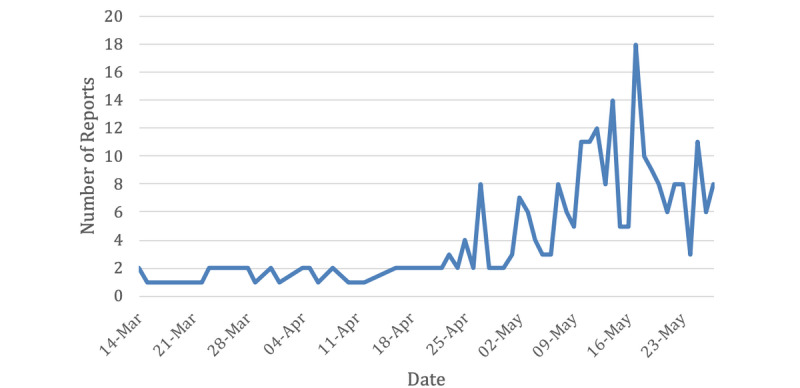
Number of daily collected reports from Twitter (March to May 2020).

We collected 893 symptoms from 270 self-reports (as shown in Table 1). The daily number of collected tweets is highlighted in Figure 4.

**Table 1:** Number of symptoms experienced by tweeters (N=270).

Symptom count	Number of reports, n (%)
0	36 (13)
1	19 (7)
2	35 (13)
3	65 (24)
4	50 (19)
5	35 (13)
6	11 (4)
7	8 (3)
8	5 (2)
9	3 (1)
10	3 (1)

Table 1 indicates that most tweeters experienced between 2 to 5 symptoms, whereas 13% (n=36) of the reported cases were asymptomatic. Table 2 lists the frequency of each symptom ordered from the most prevalent to the least. Only fever was experienced by nearly 60% (n=139) of users. The frequency of symptoms appears to be consistent across male and female patients (correlation coefficient=0.966). Further, Table 3 lists the top 8 symptoms in the order of their appearance (ie, first, second, and third rank); this refers to the 8 most common symptoms that were ranked as the first, second, or third symptom to occur in the collected self-reports. Fever and headache were commonly the first reported symptoms. The top 4 symptoms that coincided with fever were headache (n=33, 23.7%), cough (n=20, 14.4%), anosmia (n=19, 13.7%), and ageusia (n=17, 12.2%). Other symptoms occurred at a relatively lower frequency with fever. Table 4 lists the top 8 common symptoms for Saudi Arabia and Kuwait, which accounts for 81.2% (n=190) of the reports. The symptoms had a correlation coefficient of 0.835 between the 2 countries.

**Table 2:** Most common symptoms reported by users.

Symptom	All users (n=234), n (%)	Male (n=171), n (%)	Female (n=63), n (%)
Fever	139 (59)	98 (57)	41 (65)
Headache	101 (43)	68 (40)	33 (52)
Anosmia	91 (39)	63 (37)	28 (44)
Ageusia	72 (31)	51 (30)	21 (33)
Fatigue	68 (29)	54 (32)	14 (22)
Cough	62 (26)	48 (28)	14 (22)
Sore throat	42 (18)	30 (18)	12 (19)
Dyspnea	33 (14)	26 (15)	7 (11)
Diarrhea	27 (12)	22 (13)	5 (8)
Runny nose	23 (10)	17 (10)	6 (9)
Arthralgia	16 (7)	10 (6)	6 (9)
Chest pain	15 (6)	13 (8)	2 (3)
Back pain	14 (6)	11 (6)	3 (5)
Anorexia	14 (6)	11 (6)	3 (5)
Body ache	12 (5)	8 (5)	4 (6)
Nausea	12 (5)	8 (5)	4 (6)
Osteodynia	11 (5)	8 (5)	3 (5)
Dry throat	9 (4)	6 (3)	3 (5)
Myalgia	9 (4)	7 (4)	2 (3)
Dizziness	8 (3)	6 (3)	2 (3)
Chills	7 (3)	5 (3)	2 (3)
Nasal congestion	7 (3)	4 (2)	1 (2)
Sinusitis	7 (3)	3 (2)	4 (6)

**Table 3:** The top 8 symptoms, with a first, second, and third rank, as reported by users.

Number	First	Second	Third
1	Fever	Fever	Fever
2	Headache	Headache	Headache
3	Anosmia	Fatigue	Anosmia
4	Fatigue	Cough	Ageusia
5	Cough	Ageusia	Fatigue
6	Sore throat	Anosmia	Cough
7	Runny nose	Sore throat	Anorexia
8	Diarrhea	Arthralgia	Dyspnea

**Table 4:** The top 8 common symptoms for Saudi Arabia and Kuwait.

Symptom	Saudi Arabia (n=110), n (%)	Kuwait (n=80), n (%)
Fever	65 (59)	45 (56)
Headache	42 (38)	38 (48)
Anosmia	46 (42)	21 (26)
Ageusia	36 (37)	19 (24)
Fatigue	31 (28)	19 (24)
Cough	21 (19)	19 (24)
Sore throat	22 (20)	11 (14)
Dyspnea	14 (13)	11 (14)

**Figure 5:**
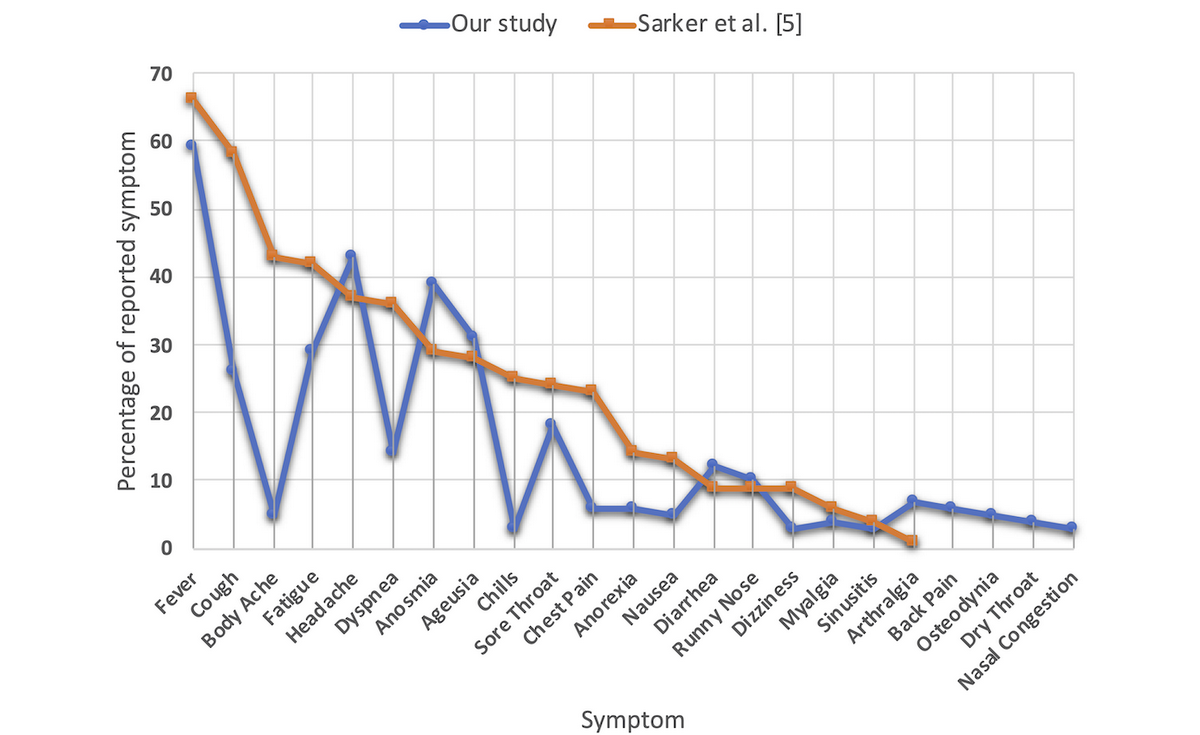
A comparison between symptom prevalence in our study and Sarker et al [5] (correlation coefficient=0.72).

Finally, we compared the symptom prevalence of our study to the one provided by Sarker et al [5], in order to assess similarities and differences in COVID-19 symptoms experienced by different populations. As seen in Table 5 and Figure 5, our findings complement those of Sarker et al [5] (correlation coefficient=0.72).

**Table 5:** Comparison of common symptoms found in this study and in Sarker et al [5].

Symptom	Our study (n=234), n (%)	Sarker et al (n=171), n (%)
Fever	139 (59)	113 (66)
Headache	101 (43)	64 (37)
Anosmia	91 (39)	49 (29)
Ageusia	72 (31)	48 (28)
Fatigue	68 (29)	72 (42)
Cough	62 (26)	99 (58)
Sore throat	42 (18)	41 (24)
Dyspnea	33 (14)	62 (36)
Diarrhea	27 (12)	15 (9)
Runny nose	23 (10)	16 (9)
Arthralgia	16 (7)	2(1)
Chest pain	15 (6)	39 (23)
Back pain	14 (6)	—^a^
Anorexia	14 (6)	23 (14)
Body ache	12 (5)	73 (43)
Nausea	12 (5)	19 (13)
Osteodynia	11(5)	—
Dry throat	9 (4)	—
Myalgia	9 (4)	10 (6)
Dizziness	8 (3)	15 (9)
Chills	7 (3)	43 (25)
Nasal congestion	7 (3)	—
Sinusitis	7 (3)	7 (4)

^a^Not applicable.

## Discussion

### Principal Findings

This work identified common COVID-19 symptoms from Arabic personal reports on Twitter. These findings complement the results of other recent studies [5,6,9] that focused on tweets in English or specific demographic groups. This study was carried in a way to report not only the symptoms but their timeline as narrated by users. Social networks have become the de facto communication channel for a large number of people. Many individuals worldwide write, interact, or even just browse social network content countless times a day. Social networks have the property of being continuously updated with new information provided by other global citizens. As such, it is crucial to monitor their content to identify health issues [13,14]. One potential benefit of analyzing social networks is understanding COVID-19 symptoms and identifying people at high risk [7].

Anosmia being one of the top 3 reported symptoms, mentioned in 39% of reports, was a surprising result in our study. Several tweeters complained about the longevity of anosmia. Our sample size is still relatively small to make any sound judgment in this regard. However, recent clinical studies have reported finding anosmia in 35.7% of mild cases of COVID-19, which is relatively close to our estimation from the tweets examined in this study [15]. In fact, the number of self-reports reflects the testing capacity of different countries. As of June 9, 2020, Saudi Arabia had completed almost 1 million tests, and Kuwait had carried out more than 350,000 tests [16].

It is worth noting that some users experienced weight loss due to COVID-19; one user claimed losing 20 kg due to the disease. Another interesting observation is that several users experienced what they described as a short-term mild fever for a couple of hours only. Quitting smoking was a positive outcome of COVID-19, per one user’s tweet. We were surprised by some users in early April claiming to be positive for COVID-19, which later turned out to be an April Fool’s Day prank. These findings prompt further study into how different communities react to a pandemic and how it affects their lives.

### Limitations and Future Works

Several limitations need to be acknowledged. Self-reports from Egypt, the largest Arabic country with almost 100 million people, were inadequately represented in this study. This could be attributed to factors such as Egypt’s preference for other social media platforms (eg, Facebook), as well as differing dialects and use of local idioms.

Our study tracked 2 widely used keywords to identify Arabic patients with COVID-19 on Twitter, followed by a manual extraction of symptoms. More complex keywords could reveal additional interesting patterns about symptoms. Furthermore, we used Modern Standard Arabic (MSA) keywords to obtain a general view of Twitter content in Arabic. It is, however, well noted in the literature that many Arabic users write in their own local dialect on social media. Hence, it is helpful to consider not only keywords in the MSA form but also keywords that are tailored toward different Arabic dialects to better capture tweets on COVID-19 symptoms written in Arabic. This may explain why Egypt was underrepresented in this study. Therefore, a multidialect COVID-19 Arabic dictionary and an natural language processing–based algorithm to detect and analyze tweets in Arabic need to be developed; establishing a comprehensive medical dictionary for different local Arabic dialects is an important line of research during the coronavirus pandemic [17].

We have extracted symptoms from users who likely underwent a screening test and, hence, tweeted based on its result; however, we do not have confirmation of testing. In this study, we have not used other COVID-19 sources; specifically, studying personal reports in Arabic from both Facebook and Twitter would have enhanced study results.

The noticeable increase in May reports compared to other months demonstrates the importance of developing a real-time surveillance system based on the symptoms reported in Twitter posts in Arabic. It also suggests further studies of information sharing behaviors in different communities and across different demographic groups (ie, users grouped by age, gender, geolocation, etc) are needed [18].

One interesting observation from our analysis is related to gender distribution. Approximately 25% of the collected reports came from female users. This could be due to several reasons. One reason could be the presence of more male Arabic patients with COVID-19 than female ones; however, we are not aware of any reliable source to support this claim. Nevertheless, in Saudi Arabia, cases reported by males consistently outnumbered those reported by females in April and May 2020 [19]. Further insights and studies are needed to investigate the gender differences in information sharing behaviors and analyze whether there is any notable difference in how male and female Arabic users disclose health information on social media.

Privacy is one of the key issues that needs to be addressed before utilizing social media for public health surveillance. Apart from each network’s privacy policy, there exists no global concensus on what to disclose when collecting health information from social media networks. Some attempts in the literature have suggested best practices to follow when collecting health information from Twitter [20]. Such practices include, among other things, avoiding quoting directly from users’ tweets and mentioning users’ IDs. Moreover, some social media sites have updated their privacy policy to further control content redistribution. For instance, Twitter’s updated policy permits redistribution of only the tweets’ ID and not their content verbatim to third parties [21].

### Conclusion

This study identified the most common self-reported COVID-19 symptoms from tweets in Arabic. Our findings demonstrated that fever, headache, and anosmia are the 3 most common symptoms experienced by users, and we presented symptom prevalence for two of the largest clusters found in our tweets database (Saudi Arabia and Kuwait).

## Abbreviations

MSA: Modern Standard Arabic
