# Correction: Identifying and Ranking Common COVID-19 Symptoms From Tweets in Arabic: Content Analysis

**DOI:** 10.2196/26446

**Published:** 2020-12-14

**Authors:** Eisa Alanazi, Abdulaziz Alashaikh, Sarah Alqurashi, Aued Alanazi

**Affiliations:** 1 Center of Innovation and Development in Artificial Intelligence Umm Al-Qura University Makkah Saudi Arabia; 2 University of Jeddah Jeddah Saudi Arabia

In “Identifying and Ranking Common COVID-19 Symptoms From Tweets in Arabic: Content Analysis” (J Med Internet Res 2020;22(11):e21329) one error was noted.

This article was originally published with a layout error causing two inline figures representing Arabic keywords to appear as regular figures in the first paragraph of the Methods section. The sentence where these inline figures should have appeared read as follows:

First, we searched Twitter for personal reports of COVID-19 from March 1, 2020, to May 27, 2020, using 2 Arabic keywords Inline Figure 6 and Inline Figure 6, which translate roughly to “I have been diagnosed.”

This has been corrected to place the two inline figures within this sentence instead of the words “Inline Figure 6” in each instance.

The correction will appear in the online version of the paper on the JMIR Publications website on December 14, 2020, together with the publication of this correction notice. Because this was made after submission to PubMed, PubMed Central, and other full-text repositories, the corrected article has also been resubmitted to those repositories.

